# Biological evaluation of Safrole oil and Safrole oil Nanoemulgel as antioxidant, antidiabetic, antibacterial, antifungal and anticancer

**DOI:** 10.1186/s12906-021-03324-z

**Published:** 2021-05-29

**Authors:** Ahmad M. Eid, Mohammed Hawash

**Affiliations:** Department of Pharmacy, Faculty of Medicine and Health Sciences, An-Najah National University, Nablus, P.O. Box 7 Palestine Nablus,

**Keywords:** Safrole oil, Antioxidant, Antidiabetic, Antimicrobial, Anticancer and Nanoemulgel

## Abstract

**Background:**

Safrole is a natural compound extracted from various plants, and has shown various biological activities. The current study aimed to investigate the antioxidant, antidiabetic, antimicrobial, and anticancer activity of safrole oil and to study the influence of safrole nanoemulgel on these activities.

**Methods:**

The antioxidant and antidiabetic in-vitro assays were conducted using standard biomedical methods. The safrole oil nanoemulgel was developed using a self-emulsifying technique. Then the antimicrobial activity of the safrole oil and safrole nanoemulgel were performed on different microbial species, and cytotoxicity was determined against Hep3B cancer cell lines using the MTS assay.

**Results:**

Safrole oil showed moderate antioxidant activity compared with standard Trolox, with IC_50_ value 50.28 ± 0.44 and 1.55 ± 0.32 μg/ml, respectively. Moreover, it had potent α-amylase inhibitory activity (IC_50_ 11.36 ± 0.67 μg/ml) compared with Acarbose (IC_50_ value 5.88 ± 0.63). The safrole nanoemulgel had pseudo-plastic behaviour, droplet sizes below 200 nm, a polydispersity index (PDI) below 0.3, and a zeta potential of less than − 30 mV. Safrole oil has potential antimicrobial and anticancer activities, and these activities were improved with safrole nanoemulgel.

**Conclusion:**

The safrole oil may be applied for the prevention and treatment of oxidative stress, diabetes, different microbial species and cancer, and these activities could be improved by nano-carriers.

## Background

Nanotechnology has influence many areas of science in recent years [1]. The word “nano” means miniature size or very small structure [2], and nanotechnology can be defined as the employment of science using different tools and systems to make extremely small structures. Nano-dosages of pharmaceuticals are different from the bulk forms, which have specific biological and physiochemical properties [3, 4]. This technology has many advantages in various science fields, especially in the production of dosage forms and the pharmaceutical industry; it is used to make the medication more effective, to deliver drugs to a specific site, to improve the stability of dosage forms, and to produce cheaper dosage forms with less side effects, which leads to improve patient compliance [5–7]. In addition, the different types of nano-dosages help to boost drug stabilisation and drug absorption and increase the passage of poorly soluble drugs into the cell, which is typically due to their large surface area. The use of nano-dosage types has ensured substantial progress in the development of drug delivery systems, some of which include nanoemulgels, nanoemulsions, and liposomes [8–11].

A nanoemulsion is a drug delivery system consisting of emulsified oil and water, with an average droplet size of 5–200 nm [12]. It is considered a highly kinetically stable system that prevents creaming, flocculation, and aggregation from occurring [13, 14]. It can deliver both hydrophilic and lipophilic drug agents and can be administered via different routes, such as topical, oral, and parenteral routes [15]. An emulsion is a compound composed of at least two immiscible liquid phases, one of which is distributed into the other as globules. It is an effective dosage type for drugs with low water-solubility [16, 17]. However, emulsions have certain limitations because of some parameters, such as pH, temperature, and stability. The emulsions may also be subjected to creaming, coalescence, and flocculation [18].

Moreover, within the main collection of semi-solid preparations, gel is a clear, semi-solid dosage medium of good stability and application compared to cream and ointment. Nevertheless, it is inefficient for the supply of poorly soluble medicines [19]. To overcome these limitations of gels and emulsions, they can be combined in the form of an emulgel [20], which has high solubilising ability and permeation-enhancing properties [21]. In addition, it is easily spreadable and removable and has a pleasing appearance [22]. Furthermore, an emulgel possesses a dual-release control system from this combination of hydrogel and nanoemulsion [23, 24].

In the last few years, pharmaceutical research has focused more on natural bioactive compounds. Plant extracts or raw plants have a range of phytochemicals and bioactive components that provide synergistic therapeutic effects, which have multi-target effects in the curing of diseases [25]. However, some of these extracts have been used as anticancer agents since cancer is considered the disease with the greatest mortality [26] and as antidiabetic agents since diabetes mellitus is considered one of most common diseases [27]. The use of medicinal plants is considered one of the major approaches to the production of natural medicinal agents [28]. Recently, many researchers have investigated the different types of herbs to determine or improve their therapeutic properties or to determine their isolated compounds for use in the pharmaceutical industry and clinical practice [29, 30]. Safrole is a major component of sassafras oil and a component of several other essential oils [31]. It has differential biological activities, such as cytotoxic, analgesic and antimicrobial activities [32–34].

Several studies have been carried out on safrole derivatives and their pharmacological effects, particularly their cytotoxicity [34–36], Twenty-three naturally occurring and synthetic alkenylbenzene derivatives that are structurally related to safrole (1-allyl-3,4-methylenedioxybenzene) were tested for their hepatic-carcinogenicity in mice [37] and were classified as group 2B carcinogen extracted from betel quid-chewing, one of the major risk compounds for oral squamous cell carcinoma and hepatocellular carcinoma development [38, 39]. In this work, we tried to evaluate the safrole nanoemulsion against various biological targets.

## Methods

### Materials

Safrole oil was purchased from (Sigma-Aldrich, Germany). The carboxyvinyl polymer (Carbopol 940) was obtained from the CBC Co., Ltd., Japan. Dimethyl Sulfoxide (DMSO) was obtained from Riedel-de-haen, (Seelze, Germany). Trolox [(S)-(−)-6-hydroxy-2, 5, 7, 8-tetramethylchroman-2-carboxylic acid] and 2,2-Diphenyl-1-picrylhydrazyl (DPPH) were purchased from Sigma Aldrich (Denmark). Both surfactants Span and Tween were bought from Al-Shamas company (Palestine). DNSA 3,5-Dinitrosalicylic acid (DNSA) reagent was purchased from Sigma-Aldrich (LA, USA) Methanol and *n*-hexane were obtained from Loba Chemie (India). N-Succ (Ala) 3-o-nitroanilide (SANA) and porcine pancreatic elastase (PPE) were obtained from Sigma Aldrich, USA. The α-amylase enzyme from Sigma Aldrich (Mumbai, India), and acarbose was obtained from Sigma-Aldrich (St. Louis, USA).

### Antioxidant testing of safrole oil

The antioxidant activity of the safrole oil was evaluated using the free radical-scavenging assay. DPPH was used to measure the scavenging activity of the oil. Stock solutions of the safrole oil and trolox (as a standard reference compound) were prepared at a concentration of 1 mg/mL, from which serial dilutions were carried out (1, 2, 3, 5, 7, 10, 20, 50, 80 and 100 μg/mL). One millilitre of the stock solution and 1 ml of methanol were mixed with 1 ml of DPPH solution. The solution was then incubated in the dark for 30 min at room temperature, and the blank solution was prepared by replacing the stock solution with methanol. Trolox was used as a control, and the absorbance was measured by a UV–Visible (UV–Vis) spectrophotometer at 517 nm, which was compared with the control. The following equation was used to calculate the percentage of DPPH inhibition by the trolox standard and the safrole oil:

$$ \mathrm{DPPH}\ \mathrm{inhibition}\ \left(\%\right)=\left(\mathrm{B}-\mathrm{T}\right)/\mathrm{B}\times 100 $$

Where B is the absorbance of the blank and T is the absorbance of the tested samples. The IC50 of the antioxidant (50% Inhibition concentration) was calculated for both Trolox and the safrole oil using BioDataFit edition 1.02 [40].

### α-Amylase inhibitory effect of safrole oil

The assay for α-amylase inhibition was conducted using the process of 3,5-dinitrosalicylic acid (DNSA). Safrole oil was dissolved in a minimum of 10% DMSO and subsequently dissolved in the buffer (Na_2_HPO_4_/NaH_2_PO_4_; 0.02 M), NaCl (0.006 M) at pH 6.9 to yield 1000 μg/ml concentrations, from which the following dilution was prepared: 10, 50, 70, 100, 500 μg/ml. A 200 μl volume of the porcine pancreatic α-amylase enzyme solution (2 units / ml) was combined with 200 μl of safrole oil, and incubated at 30 °C for 10 min. Two hundred microlitres of the freshly prepared starch solution (1% in water [w/v]) was then added to each tube, and the solution was incubated for 3 min. Termination of the reaction was carried by adding 200 μl of DNSA reagent (12 g of sodium potassium tartrate tetrahydrate in 8.0 mL of 2 M NaOH and 20 mL of 96 mM of 3.5-dinitrosalicylic acid solution) and then diluted with 5 ml of distilled water and boiled in a water bath at 85–90 °C for 10 min. The mixture was cooled to ambient temperature. Then using a UV-Visible spectrophotometer, the absorbance was taken at 540 nm. The blank with 100% enzyme activity was primed with 200 μl of the buffer to replace the safrole oil. A blank reaction in the absence of the enzyme solution was similarly prepared using safrole oil at each concentration [41]. By following the steps outlined above, a positive control sample was created using acarbose, and the reaction was similar to the reaction with plant fractions mentioned above The α-amylase inhibitory activity was expressed as the percent inhibition and was calculated using the equation given below:

$$ \%\mathrm{of}\ \alpha -\mathrm{amylase}\ \mathrm{inhibition}=\left(\mathrm{B}-\mathrm{S}\right)/\mathrm{B}\ast 100\% $$

Where B: is the absorbance of the blank and S: is the absorbance of the tested sample [26].

### Preparation of the nanoemulgel

The nanoemulgel was rendered in sequential steps by incorporating the nanoemulsion in the hydrogel.

### Preparation of the nanoemulsion

Using a self-emulsifying process, safrole oil nanoemulsions were created using three different elements at different concentrations. The Safrole oil, Tween® as surfactant and Span® as a co-surfactant were the elements.

The self-emulsifying method is a combination of natural or synthetic oils, solid or liquid surfactants, and co-surfactants with isotropic activity [42], Spontaneous emulsifications when combined with aqueous agitation are the main explanation for this combination [43].

This process can be explained by the fact that the energy needed to raise the dispersion surface is less than the entropy change, which promotes dispersion. It allows the energy to create a new surface between the two points [44].

A ternary phase diagram using the three elements above was generated to refine the formulation of nanoemulsion. Using a sensitive balance, each formulation was measured and then vortexed (CLASSIC Advanced Vortex Mixer) for 2 min. The oil was then emulsified with water under gentle agitation. Finally, the physical properties have been calculated and based on these characteristics the ideal formulation has been chosen.

### Analysis of the characteristics of the nanoemulsion

A NanoBrook Omni 280,173 (Brookhaven, New York) was used to determine the polydispersity index (PDI) and droplet size of the nanoemulsion. The mean and standard deviation were measured in triplicate. The formulation that provided the smallest droplet size with the lowest PDI and the highest amount of oil was selected.

### Hydrogel formulation

The hydrogel was created by the addition of Carbopol® 940 to water and continuous mixing using a homogeniser to prepare a uniform dispersion. Subsequently, under constant mixing, 2 M NaOH was added to the hydrogel to adjust the pH to 6. The mixture underwent continuous mixing and was left for 24 h to completely gel.

### Nanoemulgel formulation

The nanoemulgel formulation was prepared by the addition of different concentrations (0.4, 0.6, and 0.8%) of the hydrogel matrix to the optimum nanoemulsion at 100 rpm for 10 min. The PDI, droplet size, and zeta potential were subsequently measured.

### Physical characterisation of the nanoemulgel

The droplet size, PDI, and spreadability, in addition to the visual appearance of the nanoemulgel formulation were inspected.

### Analysis of the zeta potential of the nanoemulgel

The zeta potential is defined as the potential that forms between the oil droplet and the liquid phase. Stable formulations have a positive or negative potential that is greater than 30 mV. A NanoBrook Omni 280,173 (Brookhaven, New York) was used to measure the zeta potential of the nanoemulgel formulation.

### Rheological measurement of the nanoemulgel

The rheological performance of the nanoemulgel formulations obtained at different Carbopol® 940 concentrations (0.4, 0.6, 0.8%) as a gelling agent was assessed at ambient temperature using a rotational viscometer (Brookfield DV1, USA). All measurements were performed in triplicate. In the shear rate range (0–100 rpm), the viscosity was calculated.

### Antimicrobial test

#### Microorganisms

The organisms used for the bacterial test were *Escherichia coli*, *Staphylococcus aurous*, *MRSA*, *Pseudomonas aeruginosa*, *Klebsiella, Proteus vulgaris* and *Enterococcus faecium*, and *Candida* was used for the fungal test.

#### Culture media

Muller Hinton agar is a bacterial crop medium containing 2 g of beef extract, 1.5 g of starch, 17.5 g of casein hydrolysate, and 17 g of agar per liter of filtered water. The media were created by combining the components and heating them with restlessness until they boiled. The media was then autoclaved at 121 °C for 20 min. Following cooling, the agar was poured onto a flat surface in sterile plates of petri, which were held in a depth of 4 °C. Sabouraud dextrose agar containing 40 g dextrose, 10 g of peptone and 20 g of agar per liter of distilled water was the medium used for fungal production. As mentioned above, the media was prepared.

McFarland turbidity standards were used to standardise the inoculums. McFarland 0.5 standard was used for turbidity comparison, which offers a turbidity similar to that of a bacterial suspension containing 1.5 × 10^8^ CFU/mL.

#### Agar well diffusion test

For inoculation of normal bacterial cultures, a plate containing Muller Hinton agar was inserted with 6-mm opening. A was negative control, B was loaded with safrole oil natural, C was filled with the safrole oil nanoemulgel and D was eventually packed with 500 mg of amoxicillin dissolved with 10 mL of water in the bacterial test. The plate was incubated at 35 °C for 24 h. The fungal assay consisted of 100,000 IU nystatin and was incubated for 48 h at 37 °C. Antimicrobial activity was measured using the diameter of the inhibition zone.

#### Cell culture and cytotoxicity assay

The RPMI-1640 media was used to culture hepatocellular carcinoma cells (Hep3B cells) and combined with 10% fetal bovine serum, 1% Penicillin/Streptomycin antibiotics and 1% l-glutamine. Cells were cultivated with 5% CO_2_ at 37 °C in a moist environment. Cells were plated on a 96-well substrate at 2.6 × 10^4^ cells/well. Various concentrations of the safrole oil and safrole oil nanoemulgel were incubated for 24 h, and after 48 h cell viability was measured in compliance with the guidance of the supplier (Promega Company, Madison, WI) by a CellTilter96® Aqueous One Solution Cell Proliferation (MTS) Assay. In brief, 20 μL of MTS solution was applied at the end of treatment per 100 μL of medium and incubated at 3 wells [45].

### Statistical analysis

The findings from safrole oil and safrole nanoemulgel were expressed as the mean ± standard deviation (SD). The T-test was used to compare pooled results. When the *p*-value was < 0.05, the statistical meaning was taken into consideration.

## Results

### Antioxidant activity of Safrole oil

**Fig. 1:**
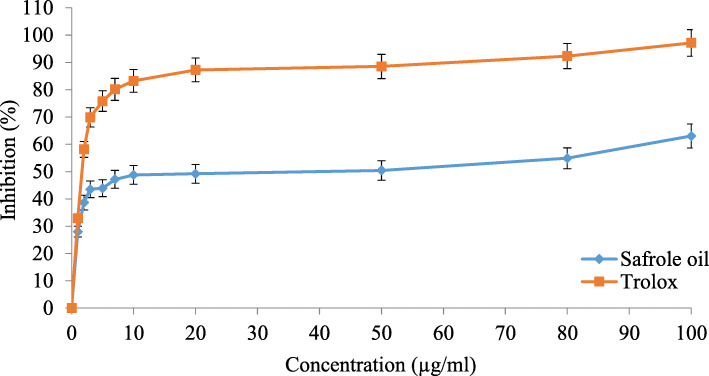
Antioxidant activity of Trolox and safrole oil

The antioxidant activity of safrole oil is related to the scavenging activity of free radicals, it was comparatively lower than the Trolox reference standard, which is known to have high antioxidant activity. However, the results shown in Fig. 1 revealed that safrole oil has antioxidant activity, with an IC_50_ of 50.28 ± 0.44 μg/mL for safrole oil and an IC_50_ of 1.55 ± 0.32 μg/mL for Trolox.

### α-Amylase inhibitory activity of Safrole oil

**Fig. 2:**
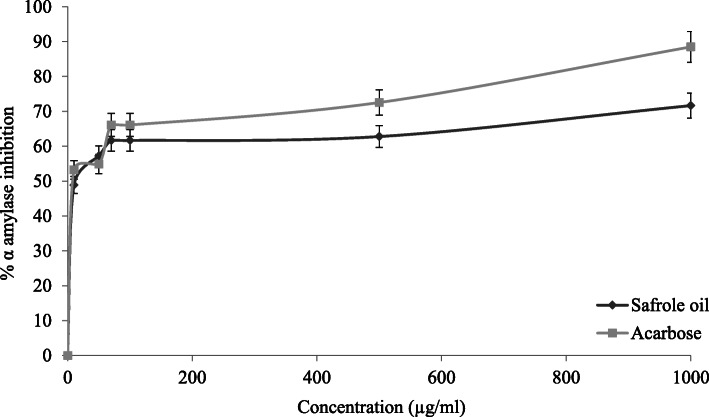
The results of α-amylase inhibition for the acarbose standard and safrole oil

The α-amylase inhibitory activity of safrole oil was determined and compared with the positive control (acarbose). Figure 2 showed that the safrole oil effectively increased the inhibition rate of the α-amylase enzyme, which has an IC_50_ values of 11.36 ± 0.67 μg/ml, in comparison with acarbose, which is the reference compound that had an IC_50_ value of 5.88 ± 0.63 μg/mL.

### Droplet size and polydispersity index of safrole oil nanoemulsion formulations

**Fig. 3:**
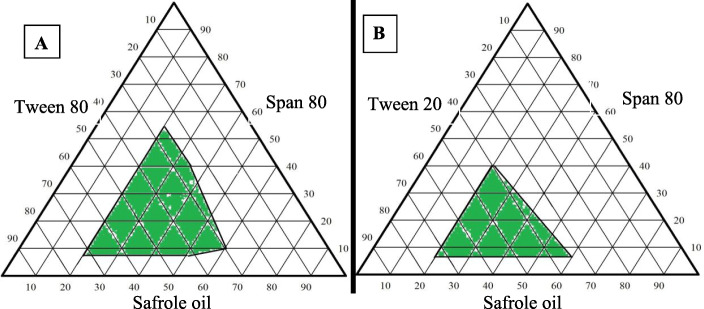
Ternary-phase diagrams of the safrole oil nanoemulsion constructed with A) Tween 80 and Span 80 and B) Tween 20 and Span 80

To choice an appropriate formulation for a nanoemulsion with droplet size below 200 nm and a PDI of less than 0.3, two separate compilations of surfactant/co-surfactant with safrole oil were utilized to create the ternary phase diagrams (Tween 80/Span 80 and Tween 20/Span 80). Figure 3 represents the phase diagrams of safrole oil nanoemulsions made using Tween80/Span 80 and Tween 20/Span 80. Nanoemulsions are those formulations with a droplet size of less than 1 μm, which are represented by the green area.

Both surfactants have shown a different behaviour during the emulsion preparation. Tween 80 was capable of producing a nanoemulsion with droplet sizes of less than 200 nm, while Tween 20 produced a nanoemulsion with a droplet size greater than 200 nm. However, the ideal nanoemulsion composition was chosen not only based on its scale but also the oil concentration and the PDI in the selected formulations, as can be seen in Table 1. Therefore, the optimum formulation of the nanoemulsion was prepared from 51.2% Tween 80, 12.8% Span 80, and 36% safrole oil, that has a droplet size of 116.17 nm and a PDI of 0.207.

**Table 1:** The selected formulation of the safrole oil nanoemulsion

Formulation No.	Tween 80 (%)	Span 80 (%)	Safrol oil (%)	Droplet size (nm)	PDI
1	64	16	20	197.49	0.266
2	51.2	12.8	36	116.17	0.207
3	57.6	6.4	36	184.83	0.327

### Influence of various Carbopol concentrations on droplet size and the polydispersity index of safrole oil nanoemulgel

**Fig. 4:**
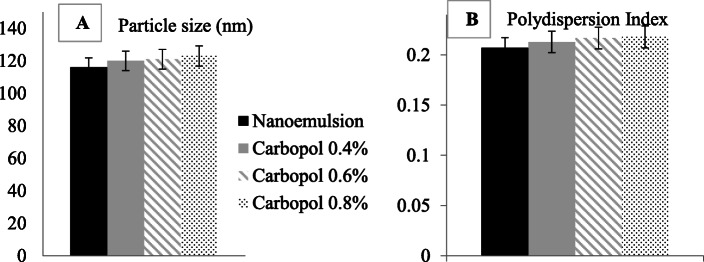
**a** Droplet size and **b** polydispersity index (PDI) of the initial safrole oil nanoemulsion and the safrole oil nanoemulgel formulations containing different concentrations of Carbopol 940 (0.4, 0.6, 0.8%)

The findings regarding the average droplet size that the developed drugs are on the sub-micron scale with a low PDI, indicating a narrow distribution of droplet size. The findings regarding the optimum formulation for the nanoemulsion were compared with the results of nanoemulgel formulations obtained with a mean droplet size and PDI. Figure 4**a** shows a comparison of the average droplet size with nanoemulsion (initial) and nanoemulgel formulations with varying concentrations of Carbopol 940. Figure 4**b** displays the size distribution of nanoemulgel formulations containing safrole oil.

### Measurement of the zeta potential of the safrole nanoemulgel

**Fig. 5:**
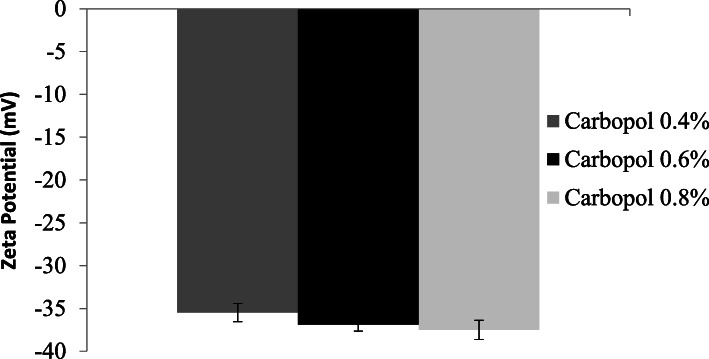
Mean zeta potential of the safrole oil nanoemulgel formulations containing different Carbopol 940 concentrations

Depending on the zeta potential test, it became apparent that all nanoemulgel formulations with safrole had a zeta potential below 30. The results are reported in Fig. 5.

### Measurement of the rheological behaviour of safrole oil nanoemulgel formulations

**Fig. 6:**
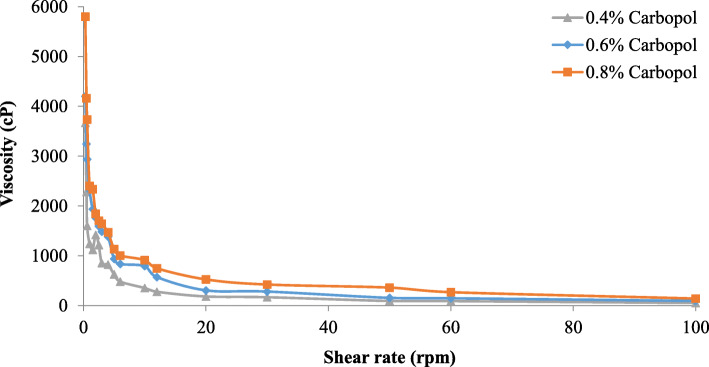
Rheological behaviour of different safrole oil nanoemulgel formulations

Typically, the flow properties of semisolid pharmaceutical products are normally regulated and measured by the rheological characteristics, which are critical to ensure the consistency and effectiveness of the formulation. The rheological analysis of the safrole nanoemulgel formulations is shown in Fig. 6. With an increase in shear rate, the viscosity decreased; therefore, the rheological behaviour of the nanoemulgel is pseudo-plastic.

### Antimicrobial of safrole oil and safrole oil nanoemulgel

The **Antimicrobial** results obtained from this work show that safrole oil has various bioactivity against the growth of microbes (MRSA, *Enterococcus faecium*, *E. coli, Klebsiella, Proteus vulgaris,* and *Staphylococcus aureus*) and as shown by the positive control (amoxicillin). The safrole oil showed no activity against *E. coli* and *Klebsiella* and partial resistance against *S. aureus* and MRSA. However, it showed good bacterial inhibition against *Enterococcus faecium, Pseudomonas aeruginosa and Proteus vulgaris*. However, it showed no activity against *E. coli* and only partial activity against *Klebsiella*. The results of the in vitro antibacterial activities were calculated in terms of the zone of inhibition diameter (mm), and they are recorded in Table 2.

**Table 2:** Antibacterial activity of the safrole oil versus the safrole nanoemulgel and amoxicillin as a positive control

Name	Negative control	Amoxicillin 750 (mm)	Safrole oil (mm)	Safrole nanoemulgel (mm)
*Enterococcus faecium* (ATCC 700221)	Resistance (No effect)	40.0 ± 0.70	11.0 ± 0.72	19.0 ± 1.40
*E. Coli* (ATCC 25922)	Resistance (No effect)	33.0 ± 0.81	Resistance (No effect)	Resistance (No effect)
*Klebsiella* (ATCC 13883)	Resistance (No effect)	18.0 ± 1.41	Resistance (No effect)	Partial resistance (Inhibition but the zone was not clear)
*Pseudomonas aeruginosa* (ATCC 9027)	Resistance (No effect)	40.0 ± 0.70	12.5 ± 0.71	20 ± 1.10
*S. aureus* (ATCC 25923)	Resistance (No effect)	42.0 ± 0.70	Partial resistance (Inhibition but the zone was not clear)	13.0 ± 1.41
MRSA (Clinically strain)	Resistance (No effect)	26.0 ± 1.41	Partial resistance (Inhibition but the zone was not clear)	14.5 ± 1.21
*Proteus vulgaris* (ATCC 8427)	Resistance (No effect)	38.0 ± 2.80	11.5 ± 0.70	17 ± 0.5
*Candida*	Resistance (No effect)	Resistance (No effect)	17.0 ± 0.80	22.0 ± 1.70

### Cytotoxic evaluation of safrole nanoemulgel

**Fig. 7:**
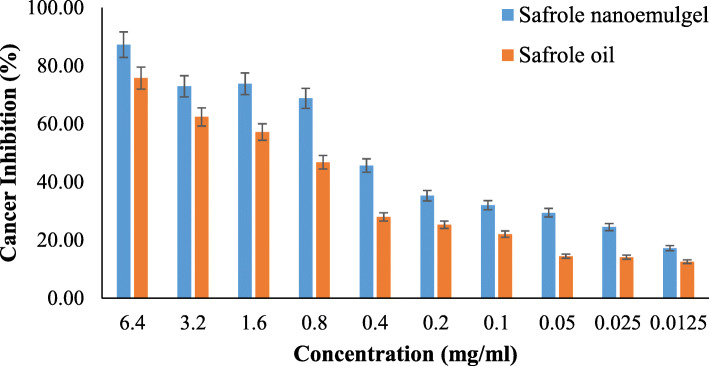
%age of cancer cell inhibition of safrole nanoemulgel vs safrole oil

The MTS assay was used to determine the cytotoxicity effect of pure safrole oil and the safrole nanoemulgel dosage form on Hep3B hepatocellular carcinoma cells. As shown in Fig. 7, different concentrations were used to investigate the cytotoxicity of the compounds. As shown in the figure, the safrole nanoemulgel showed higher cancer cell inhibition (87.25%) when compared with the safrole oil (75.72%). The IC50 value for the safrole nanoemulgel and the safrole oil was 0.31 ± 0.02 mg/mL and 1.08 ± 0.06 mg/mL, respectively. The results demonstrate an interesting outcome since the safrole oil showed activity against the cancer cell line. In addition, this activity clearly increased when safrole oil was in the form of a nanoemulgel.

## Discussion

From ancient time until now, many of the important and common pharmaceuticals have been used for a wide range of medicinal purposes. In the last two decades, the use of herbal products, known as medicinal agents, in mainstream health systems, has increased. Popular herbal remedies known as “old wives” have been clinically accepted and provide possible bioactive compounds for even the deadliest illnesses, such as cancer, atherosclerosis, diabetes, and Alzheimer’s diseases. Clearly, the medicinal herbal discovery and isolation of their active molecules actually entails large scientific research centers and also multinational pharmaceutical companies.

Medicinal plants are known to be important sources of natural antioxidants in traditional medicine [46]. Antioxidants are molecules that suppress or prevent free radicals from causing oxidation damage. They can be categorised according to the inputs from a biological source, such as enzymes, animals, insects, bacteria and chemical source. Antioxidants are usually available in our body to defend our cell, and their deficiency can induce cell harm in various forms depending on the nature of oxidation risk, such as carcinogenicity, mutagenicity, and allergy. Several of the phytogenic compounds exhibit antioxidant function at different levels, such as polyphenolic compounds, which decrease thrombosis [47, 48].

In a study conducted by Salleh and Ahmed, the authors reported safrole to be the precursor of many products, such as *Piper auritum*, which showed antioxidant activity [49]. Another study carried by Salleh et al., on *C. macrocarpum* essential oil found out the safrole was the major component of this oil (54.5–59.5%) and demonstrated significant activity on DPPH [50]. Studies indicate that antioxidants usage can minimize the oxidative stress induced by the adverse effects of some drugs [51]. These indications create the potential for a new line in cancer therapy by treating them with antioxidants.

Amylase is a carbohydrate digestion enzyme with several forms, one of which is alpha-amylase, which is produced by the salivary and pancreas gland. Diabetes mellitus (DM) is a chronic condition that has become a large burden on the health system, and its prevalence has increased dramatically because of the population’s sedentary lifestyle and obesity. One approach proposed in the treatment of type II DM is to limit the ingestion of glucose into the intestine, and this is accomplished through medications that act by inhibiting the metabolic enzymes involved in the mechanism of digestion of specific glucose sugars, namely α-amylase. This results in successful outcomes in term of a reduction in the glucose levels in blood and in the treatment of the disease. Several plants demonstrate the distinct inhibitory action of this enzyme, which may be a healthy substitute and a potential source of new medicines [52]. The comparison between the IC50 of acarbose (the reference compound) and the IC_50_ of safrole oil proves that safrole oil has potent α-amylase inhibitory activity and that it could be an excellent candidate for manufacturing potential antidiabetic herbal supplements.

A ternary phase diagram was established for identifying the self-nanoemulsion regions and for choosing the suitable surfactant, and co-surfactant concentrations used in the formulation of an optimum safrole nanoemulsion. The diagram was drawn to demonstrate the best formulation for achieving droplet sizes of less than 200 nm. Due to the perfect combination of solubility, The hydrophile-lipophile balance (HLB) values, and molecular geometry, a high nanoemulsion region of safrole oil was probable, as verified by Mayer and his team [53].

Droplet size was a key factor in the success of the nanoemulsion since the rate of release and absorption could be influenced. Furthermore, because of the smaller particle size and the large interfacial region, the bioavailability improved rapidly if the droplet was less than 200 nm. The right mixture of surfactant and co-surfactant resulted in a smaller globule size, leading to a higher mechanical barrier that explained the prevention of globule aggregation [54].

The PDI is another parameter with a value that is similar to the droplet size in the nanoemulsion formula. The distribution of the droplet size is often called the homogeneity measurement distribution. As the PDI value small values, it displays homogeneity (narrow distribution of the particles size) and better emulsion with greater physical stability.

Nanoemulgels with safrole oil were made with a variety of Carbopol 940 concentrations (0.4, 0.6 and 0.8% w/w). Carbopol is a gelling agent ingredient that gives the formulation swelling properties [55]. The technique of self-emulsification was employed to emulsify the ideal nanoemulsion formula which contains Tween 80 as surfactant, Span 80 as a co-surfactant, oil and distillation water. The nanoemulgel was formulated by continuously agitation of nanoemulation with the Carbopol 940 hydrogel. The viscosity, droplet size and size distribution of nanoemulgel formulations were calculated.

There was no substantial variation between the size of the droplets and the PDI of the safrole oil in the form of a nanoemulsion or when it was converted to a nanoemulgel. The measured stability is defined as the magnitude of the zeta potential in the submicron preparation. When the droplets have high negative and positive zeta potential, the droplets will repel each other, and if the values are low, there is dispersal instability since they are not stopped by force.

There is a boundary line between unstable and stable dispersions, which is usually + 30 or − 30 mV [56]. The zeta potential of the droplets was considered stable if it was more than + 30 mV or less than − 30 mV. The zeta potential of the safrole nanoemulgel formulations varied from − 35 to − 40 mV because of the nature of non-ionic surfactants, which reinforce the device by forming a layer around its surface. There were no significant variation between the results of the safrole nanoemulsion and the nanoemulgel, therefore, the quality of nanoemulgels developed was not impaired [6].

In semisolid preparations, rheological measurements are important for mechanically describing the system (flow properties) and for controlling the consistency, which is necessary to guarantee the performance and durability of the formulation. A higher viscosity can affect the release of drugs and their bioavailability due to the decreased drug delivery from the vehicle [57]. The findings revealed that the viscosity increases as the concentration of Carbopol increases, and the shear rate also decreased. The rheological nature of the safrole nanoemulgel was pseudo-plastic, i.e., the viscosity decreased as the shear rate increased.

The safrole nanoemulgel displayed a higher and enhanced zone of inhibition compared to the pure oil antibacterial action. There are many reasons behind these findings. First, since the nano-droplets are small in size and have a huge surface, they result in greater penetration and action [58]. Marslin et al. mentioned the same findings; he studied silver nanoparticles of *Withania somnifera* cream on microbial growth. He found an increase in *Withania somnifera* cream silver nanoparticle penetration, which led to growth inhibition. Furthermore, the packaging of nanoparticles in bacteria increases the concentration of medicines that enter bacteria by increasing the contact between medicines and bacteria [59]. In 2019, Eid et al. reported an improvement in the antimicrobial activity of fusidic acid and a sodium fusidate nanoemulgel, which related to their nano-size as well as to an increase in the drug’s residency time by the bacteria lead to more penetrating effect [60].

The cytotoxicity test is the way certain medications and chemicals can be used to evaluate their impacts on cancer cells in different action mechanisms and on which cell lines can act as a future treatment for cancer. The cytotoxic activity of safrole oil and the safrole nanoemulgel was tested against Hep3B cancer cells (Hep3B cells are derived from liver cancer). It is known to be the most common type of primary liver cancer, and it is the leading cause of cancer-related mortality worldwide [29, 61].

A study conducted by Catalan and his team on nine derivatives from safrole assessed the antiproliferative effect using different human cell lines. Some safrole derivatives demonstrated better antiproliferative effects than the parent compound on breast cancer cell lines (MCF-7) and a colorectal cancer cell line (DLD-1). These findings support the findings of the current study since safrole showed anticancer activity on different cancer cell lines [62]. In another study carried out by Song et al. on safrole from Cinnamomum longepaniculatum leaf and its activity against cancer cell growth on human hepatoma cell line (BEL-7402). This study showed that safrole significantly suppressed cell proliferation in a dose-dependent manner [63].

The techniques of nano-formulation in relation to conventional drug delivery systems are proposed for promising alternatives since these include exclusive drug supplies. These findings were proved by several studies, Taghipour et al., conducted a study on the anticancer activity of polyphenol nanoformulations as bioactive agents and concluded that the advantages of natural polyphenol against cancer can be potentiated through nanonisation through multiple pathways. The nanoformulations led to increased antineoplasty, higher intracellular polyphenol concentration, slow and long-term drug release, and improved proapoptotic activity against tumour cells [64].

## Conclusion

The present study demonstrated the potential antidiabetic, antioxidant, antimicrobial, and anticancer activities of safrole oil. It can be concluded that safrole oil has potent α-amylase and oxidant inhibitory activity compared to acarbose and Trolox. In addition, the safrole oil nanoemulgel was prepared using the self-emulsifying technique with droplet sizes below 200 nm and showed high stability, as indicated by its low zeta potential and narrow size distribution. The safrole nanoemulgel improved the activity against different microbial species when compared with pure safrole oil. Moreover, regarding the cytotoxicity assay, safrole oil was tested against a human cancer cell line (Hep3B) and showed significant cytotoxic activity against hepatocellular carcinoma cells, and this activity improved significantly when safrole oil was used in the form of a nanoemulgel. This study suggests that herbal nano-formulations containing safrole have the potential to treat oxidative stress and diabetes and can be used for cancer treatments. They can also be utilised for the development of herbal medicines or food supplements for commercial pharmaceutical use.

## Data Availability

The datasets used and/or analysed during the current study are available from the corresponding author on reasonable request.
